# Assessment of ten trace elements in umbilical cord blood and maternal blood: association with birth weight

**DOI:** 10.1186/s12967-015-0654-2

**Published:** 2015-09-07

**Authors:** Lorena Bermúdez, Consuelo García-Vicent, Jorge López, Maria Isabel Torró, Empar Lurbe

**Affiliations:** Department of Pediatrics, Consorcio Hospital General, University of Valencia, Avda. Tres Cruces s/n, 46014 Valencia, Spain; CIBER Enfermedades Raras, Instituto de Salud Carlos III, Madrid, Spain; CIBER Fisiopatología de Obesidad y Nutrición (CB06/03), Instituto de Salud Carlos III, Madrid, Spain

**Keywords:** Trace elements, Umbilical cord blood, Maternal blood, Copper, Birth weight

## Abstract

**Background:**

Trace elements are an essential nutritional component for humans and inadequate tissue-concentrations may have a significant effect on fetal size.

**Objective:**

To measure ten trace elements in blood samples from mothers and their newborns, and assess their association with anthropometric characteristics at birth. The effects of other factors on fetal growth, such as biologic characteristics of the infant and mother, were analysed.

**Methods:**

A cross-sectional study was conducted in the Hospital general, University of Valencia, Spain. Healthy pregnant women, and their full-term infants were selected (*n* = 54 paired samples). Infants were grouped according to birth weight: small for gestational age (SGA n = 11), appropriate (AGA n = 30), and large (LGA n = 13). Anthropometric and biologic characteristics of the infant and mother were recorded. Levels of ten essential elements: arsenic (As), barium (Ba), cobalt (Co), copper (Cu), chrome (Cr), iron (Fe), magnesium (Mg), manganese (Mn), selenium (Se) and zinc (Zn), in maternal and cord plasma samples were determined. Samples were obtained from the umbilical cord immediately after delivery and the samples of their mothers were drawn at 2–4 h after delivery.

**Results:**

The analysis identified that cord blood Cu (p = 0.017) and maternal blood Ba and Mg (p = 0.027 and p = 0.002, respectively) concentrations were significantly higher among SGA infants compared to AGA and LGA infants. A multiple linear regression analysis showed that increased umbilical cord Cu concentration (adjusted β −146.4 g, 95 % CI −255 to −37.7; *p* = 0.009), maternal smoking during pregnancy (adjusted β −483.8 g, 95 % CI −811.7 to −155.9; *p* = 0.005), shorter gestational age (adjusted β 350.1 g, 95 % CI 244.5 to 455.8; *p* = 0.000), and female sex (adjusted β −374 g, 95 % CI −648 to −100; *p* = 0.009) were significantly associated with decreased birth weight. Maternal anaemia was positively associated with birth weight (adjusted β 362 g, 95 % CI 20.8 to 703.1; *p* = 0.038). No significant associations were found between maternal trace elements and birth weight in multivariate analysis.

**Conclusions:**

We did not observe significant associations of cord blood trace elements other than Cu and maternal trace elements with birth weight in the multivariate analyses.

**Electronic supplementary material:**

The online version of this article (doi:10.1186/s12967-015-0654-2) contains supplementary material, which is available to authorized users.

## Background

The knowledge gained from several studies has demonstrated that intrauterine life plays a key role in the development of chronic disease in adulthood. The concept suggests that early life conditions can “program” the fetus for a spectrum of adverse health outcomes [1–6]. Fetal development depends mainly on genetic makeup, maternal nutrition and fetoplacental circulation [7]. This development is especially susceptible to the effects of environmental risk factors that disrupt the processes during a critical window of vulnerability [8, 9].

While trace elements are essential for humans, their specific roles are not well understood, especially in the prenatal period. Even though their quantities are very small in body tissue, they are involved in various biochemical pathways and are critical to the performance of certain functions that are necessary to sustain life [10]. Inadequate tissue-concentrations are associated with abnormal functioning and disease and therefore may have a significant impact on the physiological changes and requirements of the mother and fetus during pregnancy [7, 11].

In previous studies different concentrations of trace elements such as arsenic (As), iron (Fe), manganese (Mn), selenium (Se), copper (Cu), and zinc (Zn), have been found in maternal blood, placenta or umbilical cord, showing an inconsistent association with the anthropometric characteristics of the newborns [12–20]. Several factors may contribute to the discrepancies in those results from previous studies, such as heterogeneity in populations, diversity of the study designs and techniques used, among others.

Up to now studies have focused on evaluating the effects of small number of trace elements on fetal growth [16, 20, 21]. The aim of the present study was to measure ten trace elements in blood samples from mothers and their newborns, and to examine their association with anthropometric characteristics at birth. In addition to the trace elements, we analyzed the effects of some well-known predictors of fetal growth such as gestational age and sex, maternal age, parity, type of delivery, maternal smoking and hemoglobin levels during the last month of pregnancy [9, 14, 20, 21].

## Methods

### Subjects and sample collection

Newborns and their mothers from the area of the Hospital general Universitario de Valencia, Spain were enrolled in a cross-sectional study over 1 year. The inclusion criteria were newborns born at term (gestational age ≥37 weeks) in the absence of perinatal illness. All mothers were healthy, and without risk factors that may determine the size of the newborn such as, hypertension, diabetes, infections, etc. Infants were excluded if chromosomal abnormalities and/or congenital malformations were detected during gestation. According to these criteria, 54 newborns and their mothers were selected.

Birth weight, length and head circumference were registered at the time of birth by delivery room staff using standard anthropometric procedures, and data were registered in the medical record of the newborns. Gestational age was calculated based on last menstrual period, ultrasound examinations during pregnancy were extracted from the medical records, and ascertained according to the Ballard method by the neonatologist.

Three groups of newborns were created based on the growth curve database of Alexander and associates [22]: small for gestational age (lower than the 10th percentile; SGA), appropriate (birth weight between the 10th and 90th percentiles; AGA), and large (higher than the 90th percentile; LGA). Fetal size was stratified due to the importance for prognosis and clinical management of newborns.

Anthropometric and biologic characteristics of the infants (weight, length, head circumference, gestational age and sex), and mothers (age, parity, type of delivery, and hemoglobin levels of the last month of pregnancy), were collected from the medical records. The mothers did not receive any nutrient supplement or any other medication that could have influenced the concentration of trace elements. Mothers whose haemoglobin was <11 mg/dl were defined as anaemic, according to the criteria of the World Health Organization [23]. The mothers were interviewed using a structured questionnaire to determine smoking during pregnancy.

Both parents were required to sign informed consent in order to participate in the study. The study was approved by the Hospital’s review board and was carried out in accordance with the Declaration of Helsinki. Both of the samples (plasma from umbilical cord of the newborns and from peripheral veins of the mothers), as well as the collected data, were stored according to the regulations dictated by the law of Biomedical Investigation of 2007 (Law 14/2007) and all applicable rules.

### Trace element measurements

Umbilical cord blood samples were collected immediately after delivery. Blood samples from the newborns were obtained through venous puncture of the umbilical cord. The samples from their mothers were drawn at 2–4 h after delivery in non-fasting conditions, but with at least 8 h between the last meal and blood sampling. All samples were collected in EDTA tubes, centrifuged at 3000 *g* for 10 min at 4 °C, the plasma was collected, aliquoted in polypropylene tubes, and stored at −80 °C and thawed before use. Amounts of 0.5–1 mL were used for analysis.

All samples were analyzed simultaneously after the collection period and the plasmatic concentration of the ten essential elements, arsenic (As), barium (Ba), cobalt (Co), copper (Cu), chrome (Cr), iron (Fe), magnesium (Mg), manganese (Mn), selenium (Se) and zinc (Zn), were determined through inductively coupled plasma mass spectrometry (ICP-MS, Cerba International Laboratories, Spain).

### Statistical analysis

Concentrations of As, Ba, Co, Cr, Mg, Mn and Se were expressed as nM/L, and Cu, Fe and Zn were expressed as µM/L. Shapiro analysis was performed to test the normal distribution of the data. Concentrations of trace elements that were normally distributed were expressed as mean [±standard deviation (SD)] whereas those that failed to satisfy criteria for normal distribution were expressed as median [interquartile range (IQR)].

Trace element concentrations among groups were compared by the nonparametric Jonckheere’s trend test. Values of *p* < 0.05 were considered statistically significant. Pearson’s correlation and Spearman rho coefficients were used to examine the relationships between birth weight, length and head circumference, as continuous variables, and trace element levels. Likewise, correlations of trace element concentrations between umbilical cord and maternal blood by group were analyzed. An outlier analysis was performed, and levels of trace elements deviating from the mean by more than 2.5 times the standard deviation were excluded. The results shown do not include outlier values. Values below the detection limit (LOD) were included in the analysis using the predetermined value of LOD.

A multivariate linear regression model was performed to identify trace elements in cord blood associated with birth weight. The relationship between maternal blood trace elements and birth weight was also analyzed. Only variables that differed with p < 0.10 in the correlation analysis were selected for multivariate linear regression analysis. All models were adjusted for known variables as predictors of fetal growth, which were determined a priori based on previous studies, including characteristics of the infants and their mothers, such as gestational age, sex, parity, maternal age, pregnancy-anaemia, and maternal smoking during pregnancy. Maternal age and gestational age were modelled as continuous variables. Other variables were modelled as binary. The *p* values of <0.05 were considered statistically significant in the final multivariate model.

## Results

### Clinical characteristics of mothers and newborns

A total of 54 newborns and their mothers were included in the study. Among the newborns, 11 (20.4 %) were qualified as SGA, 30 (55.6 %) as AGA and 13 (24.1 %) as LGA. Even though mothers of newborns regardless of birth weight were invited to participate in the study, those with infants SGA or LGA, were more interested in participating. This explains the birth weight distribution, which is not representative of the general population. The general characteristics of mothers and their newborns in the three groups are shown in Table 1. As was expected, significant differences between girls and boys in birth weight, length, and head circumference were observed. Therefore, all comparisons among newborn groups were adjusted by sex. There were no significant differences among groups in terms of maternal age, parity, gestational age at delivery, anemia or type of delivery. Maternal smoking during pregnancy of the SGA group was significantly higher than those of the AGA and LGA groups.

**Table 1:** Anthropometric and clinical characteristics of mothers and newborns grouped by standardized birth weight

	Total (n = 54)	SGA (n = 11)	AGA (n = 30)	LGA (n = 13)	*p* value
Maternal characteristics					
Age (years)^a^	30.3 (26.3–34.1)	34.97 (28.01–35.93)	28.9 (26.46–31.33)	30.72 (28.52–32.91)	NS
Vaginal delivery (%)^a^	35 (64.8)	6 (54.5)	22 (73.3)	7 (53.8)	NS
Nulliparity (%)^a^	23 (42.6)	6 (54.5)	13 (43.3)	4 (30.8)	NS
Anemia (%)^a^	10 (18.5)	1 (11.1)	5 (18.5)	4 (30.8)	NS
Smoking (%)^a^	12 (22.2)	5 (55.6)	6 (7.6)	1 (2.3)	<0.05
Infant characteristics					
Gestational age (weeks)^b^	39.5 (38.5–40.5)	39.5 (38.5–39.5)	39.5 (37.5–40.5)	40.5 (30.5–40.5)	NS
Sex (% male)^a^	29 (53.7)	3 (27.3)	20 (66.7)	6 (46.2)	<0.05
Birth WEIGHT (g)^b^	3670 (2720–3950)	2450 (2305–2680)	3620 (2747.5–3785)	4275 (3955–4400)	<0.01
Length (cm)^b^	50 (47–51.5)	46.25 (45–46.5)	50 (48–51.25)	51.75 (50.88–52.25)	<0.01
Head circumference (cm)^b^	34 (33–36)	32.50 (31–33.5)	34.5 (33–35.88)	36.25 (33.75–37)	<0.01

p value <0.05 statistical significance of the differences among groups

*NS* non-significant

^a^Fisher’s exact tests; absolute frequency and percentage

^b^Kruskal–Wallis tests; median and interquartile range (IQR)

### Trace element concentrations in umbilical cord blood

In 54 samples of umbilical cord blood, ten elements were tested. Umbilical cord blood trace element concentrations of the study population are shown in Table 2. Elements with a normal distribution were Cu, Fe, Se and Zn in contrast to As, Ba, Co, Cr, Mg, and Mn.

**Table 2:** Trace element concentrations and their limits of detection in umbilical cord and maternal plasma

Trace element	Limit of detection	Cord plasma			Maternal plasma			Cord/maternal ratio (mean ± SD)
		Percent <LOD (%)	Mean ± SD	Median (IQR)	Percent <LOD (%)	Mean ± SD	Median (IQR)	
As (nM/L)	20	0	271.1 ± 45.4	266.4 (24.2)	55.6	26.8 ±16.5	19.9 (4.9)	11.8 ± 3.7
Ba (nM/L)	72.99	1.9	3616.6 ± 3728	3801.8 (6530.4)	1.9	2398 ± 2676	92.1 (5346.3)	5.2 ± 23.9
Co (nM/L)	2.54	0	4.90 ± 1.48	3.79 (0.43)	0	10.5 ± 7.38	6.79 (9.08)	0.64 ± 0.34
Cu (µM/L)	0.03	0	4.10 ± 1.34	3.79 (0.43)	0	23 ± 8.11	22.1 (9.6)	0.19 (0.06)
Cr (nM/L)	22.85	0	69.7 ± 16.8	63.7 (25.4)	79.6	29 ± 0.98	28.8 (0)	2.43 ± 0.61
Fe (µM/L)	0.33	0	6 ± 2.23	5.75 (2.9)	1.9	5.10 ± 1.48	5.25 (1.67)	1.54 ± 1.90
Mg (mM/L)	0.16	0	0.62 ± 0.21	0.59 (0.35)	0	0.83 ± 0.18	0.79 (0.25)	0.75 ± 0.23
Mn (nM/L)	27.3	20	61.3 ± 36.3	53 (44.6)	63	31.9 ± 10.7	27.3 (1.56)	1.99 ± 1.38
Se (nM/L)	301.2	0	438.4 ± 78.6	440.1 (105.5)	0	704 ± 199.7	705.9 (201)	1.08 ± 3.02
Zn (µM/L)	0.5	0	10.6 ± 2.35	10.5 (2.64)	0	11.6 ± 2.29	11.6 (2.11)	0.96 ± 0.33

The values are expressed as mean ± SD and median with interquartile range (IQR)

*LOD* limit of detection

The detection limits and the percentage below the LOD of ten trace elements are shown in Table 2. Differences among groups were assessed with non-parametric tests. The means (SD) of trace element serum concentrations by groups are shown in Table 3. Some trace element levels in umbilical cord (Ba, Co, Cu, Mg, Mn and Se) were higher in the SGA group compared to the other ones. These differences among groups achieved statistical significance only in the concentrations of Cu (mean SGA 4.97 µM, AGA 3.79 µM, LGA 3.49 µM; *p* = 0.017). Likewise, when using an analysis model adjusted by sex, statistically significant differences were only observed in Cu levels (*p* = 0.039).

**Table 3:** Trace elements concentrations in umbilical cord plasma and in maternal peripheral blood grouped by birth weight

	Umbilical cord blood				Maternal peripheral blood			
	SGA (n = 11)	AGA (n = 30)	LGA (n = 13)	*p* value	SGA (n = 8)	AGA (n = 25)	LGA (n = 13)	*p* value
Arsenic (nM/L)	273.5 ± 24.8	274.7 ± 56.0	260.8 ± 29.3	NS	27.09 ± 17.43	28.42 ± 19.23	23.51 ± 9.04	NS
Barium (nM/L)	4061.2 ± 3205.0	3734.0 ± 3847.5	2969.5 ± 4051.1	NS	4055.5 ± 2915.1	2325.5 ± 2585.5	1521.6 ± 2419.6	<0.05
Cobalt (nM/L)	4.87 ± 1.31	4.78 ± 1.42	4.13 ± 1.8	NS	11.71 ± 5.45	11.42 ± 8.73	7.90 ± 4.96	NS
Copper (µM/L)	4.97 ± 1.62	3.79 ± 0.98	3.49 ± 0.80	<0.05	26.68 ± 11.84	22.41 ± 6.92	21.97 ± 7.64	NS
Chrome (nM/L)	65.5 ± 12.6	70.1 ± 17.0	72.60 ± 19.7	NS	28.86 ± 0.21	29.01 ± 11.12	29.07 ± 1.02	NS
Iron (µM/L)	5.80 ± 1.52	6.26 ± 2.48	5.57 ± 2.17	NS	5.3 ± 1.17	5.17 ± 1.53	4.85 ± 1.63	NS
Magnesium (mM/L)	0.66 ± 0.22	0.64 ± 0.20	0.56 ± 0.22	NS	0.94 ± 0.19	0.83 ± 0.18	0.76 ± 0.13	<0.01
Manganese (nM/L)	67.46 ± 23.13	55.85 ± 24.49	54.85 ± 35.27	NS	36.05 ± 18.33	31.24 ± 8.54	ND	NS
Selenium (nM/L)	442.0 ± 79.81	439.0 ± 84.83	433.8 ± 67.37	NS	680.9 ± 298.6	712.37 ± 160.2	701.92 ± 213.91	NS
Zinc (µM/L)	10.03 ± 2.25	10.5 ± 2.40	11.34 ± 2.33	NS	11.82 ± 2.04	11.5 ± 2.83	11.60 ± 1.09	NS

Values are expressed as mean ± SD; p value <0.05 statistical significance of the differences among groups

*NS* non-significant, *ND* not detected

Correlation coefficients were calculated between each of the ten cord blood trace elements and anthropometric characteristics in the newborns. The analysis identified an inverse association between birth weight and Mn and Cu levels (r = −0.38, *p* = 0.005; r = −0.25, *p* = 0017, respectively). Additionally length and head circumference were also inversely associated with cord blood Mn levels (r = −0.29, *p* = 0.05; r = −0.32, *p* = 0.034, respectively). Additional file 1: Table S1.

### Maternal trace element concentrations in venous peripheral blood

Concentrations of the ten elements were determined in 46 samples of maternal blood, as eight maternal blood samples were lost due to failure in the sample collection. Maternal trace elements showed a non-normal distribution with the exception of zinc. Trace element concentrations in maternal blood and the umbilical cord blood/maternal blood ratio of the study population are shown in Table 2.

In the non-parametric analysis performed among the 3 groups, differences were only present in Ba and Mg levels in maternal blood of the SGA group. Levels were significantly higher than those of the AGA and LGA groups (Ba *p* = 0.027 and Mg *p* = 0.002), showing a decreasing trend (Table 3).

Correlations between each of the ten maternal blood elements and anthropometric characteristics of the newborns were calculated. An inverse relationship was observed between birth weight and Ba, Cu, Mg and Mn levels in maternal blood (r = −0.40, *p* = 0.005; r = −0.33, *p* = 0.026; r = −0.50, *p* = 0.000; r = −0.30, *p* = 0.041, respectively). Length and head circumference were inversely associated with maternal blood Ba (r = −0.46, *p* = 0.004; r = −0.39, *p* = 0.019) and Mg (r = −0.49, *p* = 0.002; r = −0.41, *p* = 0.014). Additional file 1: Table S1.

### Relationship between newborn and maternal trace elements

**Fig. 1:**
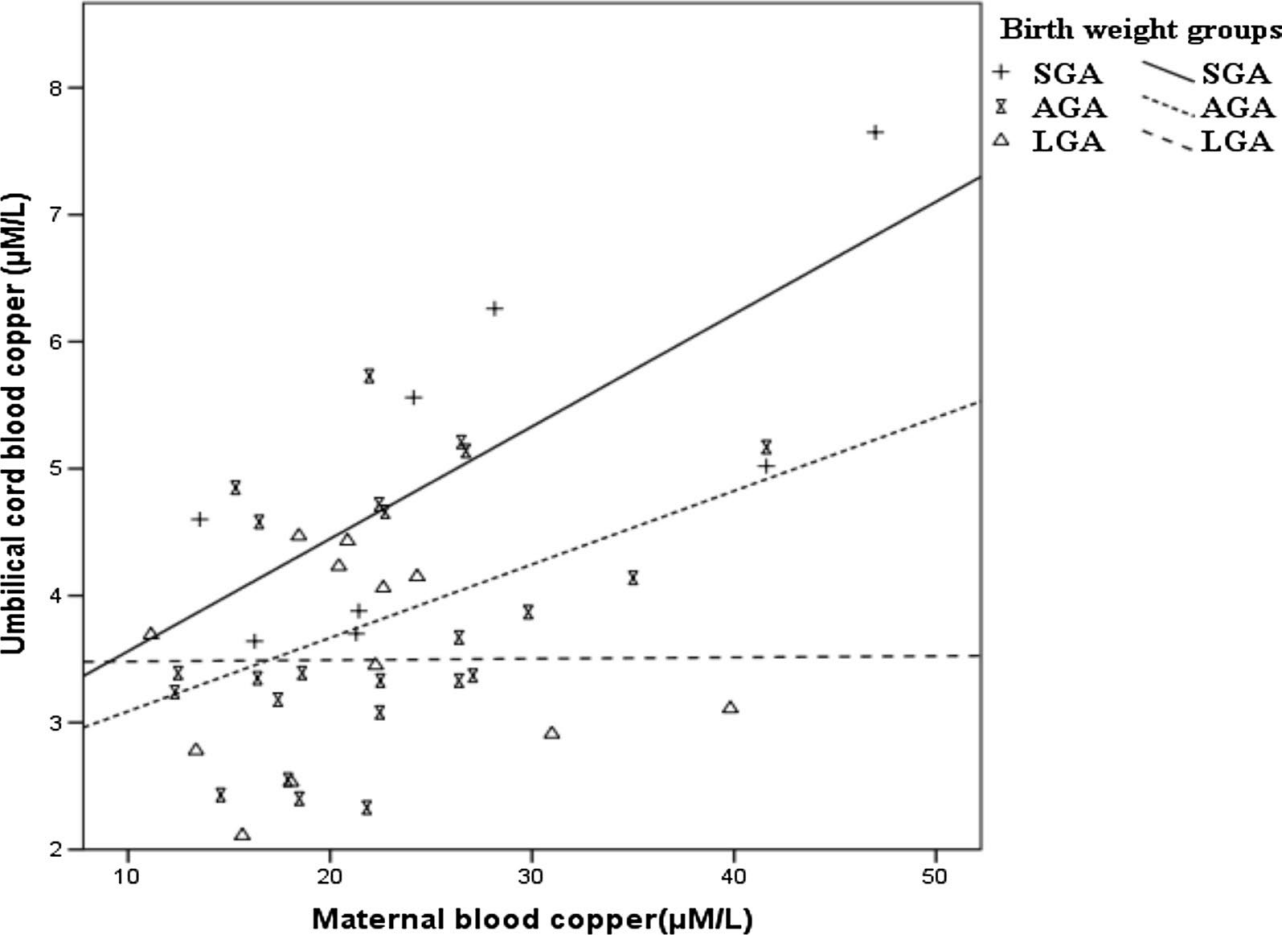
Relationship between maternal and umbilical cord blood Cu grouped by birth weight. SGA group r = 0.77, p = 0.021; AGA group r = 0.42, p = 0.043; correlation in the LGA group was not significant

A positive correlation between Cu levels in the umbilical cord and maternal blood was observed (r = 0.484; p = 0.001; Additional file 1: Table S2). Likewise, a positive relationship was found between umbilical cord and maternal blood Cu in the SGA and AGA groups (r = 0.77, *p* = 0.021; r = 0.42, *p* = 0.043, respectively). There was no significant correlation in the LGA group, Fig. 1. The correlations between the rest of the newborn and maternal trace element levels are shown the Additional file 1: Table S2.

### Multivariate analysis of factors and trace elements associated with birth weight

A multiple linear regression analysis showed that increased umbilical cord Cu concentration (adjusted β −146.4 g, 95 % CI −255 to −37.7; *p* = 0.009), maternal smoking during pregnancy (adjusted β −483.8 g, 95 % CI −811.7 to −155.9; *p* = 0.005), shorter gestational age (adjusted β 350.1 g, 95 % CI 244.5 to 455.8; *p* = 0.000), and female sex (adjusted β −374 g, 95 % CI −648 to −100; *p* = 0.009) were significantly associated with decreased birth weight. Maternal anemia was positively associated with birth weight (adjusted β 362 g, 95 % CI 20.8 to 703.1; *p*=0.038), see Table 4. The inverse correlation between the umbilical cord Mn levels and birth weight did not reach significance in the model adjusted for other variables. No significant associations were found between maternal trace elements and birth weight in the multivariate analysis.

**Table 4:** Multivariate linear regression model: R^2^: 0.62; F: 14.1; *p*: 0.000

	B	p value	95 % CI	
			Lower	Upper
(Constant)	−8975.79	0.000	−13,126.51	−4825.08
Gestational age (weeks)	350.14	0.000	244.53	455.76
Maternal smoking^b^	−483.84	0.005	−811.69	−155.98
Sex^a^	−374.00	0.009	−647.97	−100.04
Anemia^b^	361.95	0.038	20.84	703.06
Umbilical cord Cu (µM/L)	−146.39	0.009	−255.04	−37.74
Umbilical cord Mn (nM/L)	−0.98	0.640	−5.26	3.27

Independent variables: Cu and Mn umbilical cord concentrations, gestational age, maternal smoking, sex and anemia

Dependent variable: birth weight (g). p value <0.05 statistical significance

^a^Reference group = male

^b^Variables dichotomous (0 = not or 1 = yes). Reference group = 0

The associations between the different trace elements and birth length and head circumference were examined using univariate analysis, see Additional file 1: Table S2. Height and head circumference were inversely correlated with cord blood Mn levels and maternal blood Ba and Mg levels in univariate analysis, but not with multivariate models (data not shown).

## Discussion

The main finding of the present study was that Cu was the only trace element in umbilical cord blood found to be inversely and independently related to birth weight, the highest Cu concentrations were found in the SGA group. In addition, anthropometric parameters were inversely related with Mn, and no association with birth weight was observed for the other trace elements. In maternal blood, a significant trend in Ba and Mg levels, decreasing from SGA to LGA, was observed. The study is important due to the assessment of a large number of trace element levels in healthy pregnant women and their newborns, providing relevant information about their effects on birth weight and the potential adverse health outcomes later in life.

Interestingly, Cu is a trace element that is involved in the function of several cuproenzymes that are essential for life, and their requirement increases during pregnancy [24]. Although information about the effect of trace elements, simultaneously assessed, on fetal growth is scarce [15, 24–26], a negative association between Cu concentrations in umbilical cord and maternal blood and birth weight has been previously observed [15, 25]. In these previous studies, the status of other essential trace elements, Fe, Zn, Se and molybdenum (Mo), in maternal and umbilical cord blood did not influence birth weight.

The concentrations of Cu for adequate fetal development and a possible “safe level” are still unknown in humans. Copper toxicity during pregnancy and its effects on developmental outcome have been examined in considerable detail in animal models. Excess Cu has been reported to result in growth retardation in rat fetuses [27]. A strong inverse relationship was seen between increasing Cu concentrations and larval growth of a fathead minnow model [28]. Several genes associated with growth regulation were down-regulated following exposure to high Cu concentrations [28]. Although the harmful effects of high concentrations of Cu on the birth weight of newborns have been demonstrated, it is unclear if these concentrations are the cause or consequence of intrauterine growth restriction.

Even though the observed Cu in umbilical cord blood was the only independent factor related to birth weight, a positive relationship between Cu levels in umbilical cord and maternal blood was observed. The placenta is the essential interface between maternal and fetal circulation that normally maintains the proper balance of the metabolic needs of the fetus. However, it can be irreversibly damaged by oxidative stress, losing adequate functioning [29]. The role of the placenta in the transfer of some toxic elements from mother to fetus during gestation has been demonstrated [30]. The presence of a disrupted placental barrier may contribute to excessive Cu transport from the maternal blood to fetal circulation.

More recently there has been a focus on the possible effects of Mn on fetal development and pregnancy outcome. Manganese is an essential mineral nutrient in humans and other animals and is required for normal amino acid, lipid, protein, and carbohydrate metabolism. However, Mn exposure also has the potential to produce toxicity [18, 21, 31, 32]. The primary source of Mn is through diet, but exposure may also occur environmentally because of its abundance in the earth’s crust and it commonly being found in the air, water, and soil [33]. The effect of prenatal Mn levels on neonatal growth remains unclear [21, 34].

Studies have provided information concerning the relationship between Mn and birth weight [13, 14, 16, 20, 34]. Vigeh et al. [16] observed significantly lower levels of Mn in mothers who delivered infants with intrauterine growth retardation compared to mothers who delivered AGA babies. Conversely, Mn concentrations in cord blood were significantly higher in infants with intrauterine growth retardation. Zota et al. [20] performed nonlinear spline and quadratic regression models to test the hypothesis of an inverted U-shaped relationship between Mn levels and birth weight. These investigators found no association between umbilical cord blood Mn and infant birth weight in adjusted multivariate linear regression models. However, they did find a non-linear relationship between maternal blood Mn and infant birth weight [20]. These findings are similar to those recently found by Chen et al. [13] and Eum et al. [14], suggesting that both lower and higher Mn exposure is associated with lower birth weight. In a multicenter study by Yu et al., cord serum Mn was not associated with birth weight after adjusting for potential confounders [34]. However, there was a linear relationship between cord serum Mn and birth length. In the present study a clear association between Mn in umbilical cord blood and birth weight, length and head circumference was present in the univariate analyses, even though it was not found to be an independent factor.

The results of the study demonstrate that Ba and Mg in maternal blood are inversely and significantly related to all the anthropometric parameters of the newborns. Higher concentrations of Mg in cord blood of infants with intrauterine growth retardation, as compared with normal weight-newborns were observed by Barbosa et al. [35]. However, maternal samples were not available [35]. To the best of our knowledge, no study focused on Ba effects has been published.

In contrast, the association was practically non-existent between anthropometric parameters and the remaining trace elements, As, Co, Cr, Fe, Se, and Zn in both umbilical cord and maternal blood. In previous studies, the association varies widely between birth weight and trace elements such as Se, Zn, Fe, Cr and Mg, [15, 17, 19, 34–38]. Reasons to explain the discrepancies between studies are not clear, but may be related to the diversity of unknown factors that could contribute to fetal development, heterogeneity in populations, and non-uniformity in the study designs and techniques used.

In the present research additional information is provided such as the effect of other factors on fetal growth, i.e. maternal smoking and female sex, and shorter gestational age, which were significantly associated with decreased birth weight. Conversely the presence of anemia was associated with increased weight in newborns.

The findings of this study need to be interpreted within their strengths and limitations. Combining several trace elements in newborns and mothers, along with other variables of interest, the study analyses the association with anthropometric characteristics of the newborn, demonstrating the importance of Cu levels in umbilical cord. In spite of the fact that the exact toxicity limits of trace elements in the newborn are not exactly known, this study provides data on concentrations of Cu and its association with lower birth weight. This knowledge is important considering the known association between fetal growth and the development of chronic disease in adulthood [1, 3–6]. The study has limitations related to the small sample size, limiting the statistical power. Factors such as pre-pregnancy BMI, weight gain during pregnancy, socio-economic level and environmental risk were not included. Even though the sample is not representative of the general population, the inclusion of healthy pregnant women and their newborns along with a large number of trace elements led to a complete sample for analysis. Additionally, important indicators and predictors of maternal nutritional status were included such as maternal anemia and smoking habit. Moreover, given the cross-sectional design of the study, it is not possible to establish inference of causality with the factors analysed.

## Conclusions

The results demonstrate the importance of Cu levels in the umbilical cord on birth weight. There may be many factors acting additively or synergistically to trigger the condition of low birth weight, and perhaps low birth weight acts merely as a marker of a number of biological insults. The findings of this study suggest that Cu could be one of these factors. Certainly, more research is needed in order to better assess the role of trace elements in fetal growth.

## Additional file

Additional file 1. In the supplemental material section the results of the correlation coefficients between birth weight, birth length, head circumference and trace elements as well as the correlation coefficients between the maternal and cord serum levels of ten trace elements are presented.

## Abbreviations

As: arsenic
AGA: appropriate for gestational age
Ba: barium
Co: cobalt
Cr: chrome
Cu: copper
Fe: iron
ICP-MS: inductively coupled plasma mass spectrometry
LGA: large for gestational age
Mg: magnesium
Mn: manganese
Se: selenium
SGA: small for gestational age
Zn: zinc
