# Supplementary material for: Assessment of ten trace elements in umbilical cord blood and maternal blood: association with birth weight
Source: J Transl Med. 2015 Sep 7;13:291. doi: 10.1186/s12967-015-0654-2 (PMC4562355; doi:10.1186/s12967-015-0654-2)
Supplement: Additional file 1. — In the supplemental material section the results of the correlation coefficients between birth weight, birth length, head circumference and trace elements as well as the correlation coefficients between the maternal and cord serum levels of ten trace elements are presented. [file 12967_2015_654_MOESM1_ESM.doc]

**Supplementary material**

**Table S1. Correlation coefficients between birth weight, birth length, head circumference and trace elements**

| **Trace elements** | | **Birth weight** | | **Birth length** | | **Head circumference** | |
| --- | --- | --- | --- | --- | --- | --- | --- |
|  |  | **Correlation coefficient (*r*)** | ***p- value*** | **Correlation coefficient (*r*)** | ***p- value*** | **Correlation coefficient (*r*)** | ***p- value*** |
| **As (nM/L)** | **Umbilical cord** | -0.13 | *0.358* | -0,07 | *0.671* | -0.06 | *0.690* |
|  | **Maternal** | -0.05 | *0.722* | 0.03 | *0.868* | -0.21 | *0.230* |
| **Ba (nM/L)** | **Umbilical cord** | -0.19 | *0.167* | -0.16 | *0.299* | -0.17 | *0.270* |
|  | **Maternal** | -0.40 | *0.005* | -0.46 | *0.004* | -0.39 | *0.019* |
| **Co (nM/L)** | **Umbilical cord** | 0.18 | *0.208* | 0.24 | *0.124* | 0.47 | *0.002* |
|  | **Maternal** | -0.17 | *0.246* | -0.16 | *0.344* | -0.16 | *0.348* |
| **Cu (µM/L)** | **Umbilical cord** | -0.25 | *0.017* | -0.20 | *0.187* | -0.11 | *0.478* |
|  | **Maternal** | -0.34 | *0.019* | -0.28 | *0.096* | -0.24 | *0.157* |
| **Cr (nM/L)** | **Umbilical cord** | 0.10 | *0.468* | 0.06 | *0.715* | -0.03 | *0.852* |
|  | **Maternal** | 0.09 | *0.576* | 0.14 | *0.412* | -0.05 | *0.769* |
| **Fe (µM/L)** | **Umbilical cord** | -0.20 | *0.170* | -0.14 | *0.354* | -0.05 | *0.762* |
|  | **Maternal** | -0.04 | *0.794* | -0.04 | *0.814* | -0.19 | *0.263* |
| **Mg (nM/L)** | **Umbilical cord** | -0.20 | *0.148* | -0.13 | *0.398* | -0.15 | *0.348* |
|  | **Maternal** | -0.50 | *0.000* | -0.49 | *0.002* | -0.41 | *0.014* |
| **Mn (nM/L)** | **Umbilical cord** | -0.38 | *0.005* | -0.29 | *0.05* | -0.32 | *0.034* |
|  | **Maternal** | -0,30 | *0.041* | -0.28 | *0.091* | -0.24 | *0.169* |
| **Se (nM/L)** | **Umbilical cord** | 0.05 | *0.702* | 0.14 | *0.376* | -0.11 | *0.946* |
|  | **Maternal** | -0.10 | *0.491* | -0.24 | *0.146* | -0.33 | *0.051* |
| **Zn (µM/L)** | **Umbilical cord** | 0.08 | *0.562* | 0.07 | *0.633* | 0.03 | *0.842* |
|  | **Maternal** | -0.16 | *0.281* | -0.22 | *0.196* | -0.05 | *0.796* |

The Pearson’s correlation coefficient (*r*): Maternal trace element concentrations and Cu, Fe, Se, Zn of umbilical cord blood.
Rho Spearman’s correlation coefficient (r): As, Ba, Co, Cr, Mg, Mn of umbilical cord blood.

**Table S2 Correlation coefficients between the maternal and cord serum levels of ten trace elements**

| **Trace elements** | **Correlation coefficient (*r*)** | **p- value** |
| --- | --- | --- |
| **As** | 0.247 | 0.097 |
| **Ba** | 0,732 | 0.000 |
| **Co** | -0.025 | 0.870 |
| **Cu** | 0.484 | 0.001 |
| **Cr** | -0.316 | 0.032 |
| **Fe** | - 0.172 | 0.252 |
| **Mg** | 0.464 | 0.001 |
| **Mn** | 0.395 | 0.007 |
| **Se** | 0.020 | 0.897 |
| **Zn** | -0,010 | 0.945 |

The Pearson’s correlation coefficient (*r*): Cu, Fe, Se and Zn.
Rho Spearman’s correlation coefficient (r): As, Ba, Co, Cr, Mg, Mn
